# Early diagnosis of asthma in young children by using non-invasive biomarkers of airway inflammation and early lung function measurements: study protocol of a case-control study

**DOI:** 10.1186/1471-2458-9-210

**Published:** 2009-06-29

**Authors:** Kim DG van de Kant, Ester MM Klaassen, Quirijn Jöbsis, Annedien J Nijhuis, Onno CP van Schayck, Edward Dompeling

**Affiliations:** 1 Department of Paediatric Pulmonology, Maastricht University Medical Centre (MUMC), Maastricht, The Netherlands; 2 Department of General Practice, School for Public Health and Primary Care (CAPHRI), Maastricht University, Maastricht, The Netherlands

## Abstract

**Background:**

Asthma is the most common chronic disease in childhood, characterized by chronic airway inflammation. There are problems with the diagnosis of asthma in young children since the majority of the children with recurrent asthma-like symptoms is symptom free at 6 years, and does not have asthma. With the conventional diagnostic tools it is not possible to differentiate between preschool children with transient symptoms and children with asthma. The analysis of biomarkers of airway inflammation in exhaled breath is a non-invasive and promising technique to diagnose asthma and monitor inflammation in young children. Moreover, relatively new lung function tests (airway resistance using the interrupter technique) have become available for young children. The primary objective of the ADEM study (Asthma DEtection and Monitoring study), is to develop a non-invasive instrument for an early asthma diagnosis in young children, using exhaled inflammatory markers and early lung function measurements. In addition, aetiological factors, including gene polymorphisms and gene expression profiles, in relation to the development of asthma are studied.

**Methods/design:**

A prospective case-control study is started in 200 children with recurrent respiratory symptoms and 50 control subjects without respiratory symptoms. At 6 years, a definite diagnosis of asthma is made (primary outcome measure) on basis of lung function assessments and current respiratory symptoms ('golden standard'). From inclusion until the definite asthma diagnosis, repeated measurements of lung function tests and inflammatory markers in exhaled breath (condensate), blood and faeces are performed. The study is registered and ethically approved.

**Discussion:**

This article describes the study protocol of the ADEM study. The new diagnostic techniques applied in this study could make an early diagnosis of asthma possible. An early and reliable asthma diagnosis at 2–3 years will have consequences for the management of the large group of young children with asthma-like symptoms. It will avoid both over-treatment of children with transient wheeze and under-treatment of children with asthma. This might have a beneficial influence on the prognosis of asthma in these young children. Besides, insight into the pathophysiology and aetiology of asthma will be obtained.

**TRIAL REGISTRATION:**

This study is registered by clinicaltrials.gov (NCT00422747).

## Background

Asthma is one of the major chronic health problems in children. Worldwide, approximately 40% of all young children have at least one episode of asthmatic symptoms like wheezing, coughing, and dyspnoea [1,2]. Although asthmatic symptoms are common in preschool children, only 30% will have asthma at the age of 6 years and over. The rest of the children with recurrent respiratory symptoms is symptom-free at 6 years and does not has asthma but transient, viral associated wheeze [1,3,4]. A reliable diagnosis of asthma in young children is difficult. With the conventional diagnostic measures it is currently not possible to discriminate between "true asthma" in preschool children and children with "transient wheezing" in association with frequent viral infections. An early asthma diagnosis is important for the proper treatment of young children with respiratory symptoms. An effective therapy of asthma by means of anti-inflammatory treatment with inhaled corticosteroids (ICS) is available. This treatment has a beneficial influence on airway inflammation, respiratory symptoms, asthma exacerbations, quality of life, and lung function [5]. Probably, ICS are not very effective in children with transient wheezing which may cause unnecessary treatment with preventable costs and side-effects [6,7]. Therefore, an early diagnosis will prevent under-treatment of true asthmatics and over-treatment of transient wheezers, and will improve asthma control.

### Measuring inflammation

Although chronic airway inflammation is the most common feature in asthma, measurement of inflammation plays a small role in the diagnosis and monitoring of asthma. Currently, the 'golden standard' to measure airway inflammation is bronchoscopy with biopsy and/or bronchoaleolar lavage. However, this is far too invasive for normal routine use in (young) children. Since a non-invasive method to measure inflammation is lacking, diagnosis and management of asthma in young children are currently based on subjective clinical features and medical examination. Therefore, there is a lot of interest in non-invasive techniques to assess inflammation, especially in children.

### Inflammatory biomarkers in exhaled breath (condensate)

The last decade, non-invasive techniques are developed to assess inflammation in the airways. One of these new techniques is assessment of inflammatory biomarkers in exhaled breath. This technique is currently possible in young children, and is promising for an early asthma diagnosis and monitoring of the disease [8-10]. The most studied marker in exhaled breath is nitric oxide (NO). Elevated levels of fractional exhaled NO (FeNO) are found in both adults and children with asthma, as a consequence of up regulation of the enzyme iNOS [9]. In addition to FeNO, other gases can be measured in exhaled breath including volatile organic compounds (VOCs). Inflammation in the airways gives rise to reactive oxygen species (ROS) which can degradate cell membranes through peroxidation of lipids [11,12]. Due to degradation of cell membranes, volatile organic compounds, such as alkanes, alkane derivates, and aldehydes, are formed. Increased levels of alkanes (such as ethane and pentane), and aldehydes are described in exhaled breath during (exacerbations of) asthma [13-15].

Besides gases in exhaled breath, non-volatile compounds in exhaled breath condensate (EBC) can be measured in children [10,16-19]. EBC is collected by cooling exhaled breath in a condenser. During this non-invasive procedure, small droplets of breath condensate are formed. Besides water vapour, droplets consist of aerosol particles that are released from the epithelial lining fluid of the airways. They contain non-volatile inflammatory markers, and there is evidence that abnormalities in condensate composition reflect biochemical changes of the epithelial lining fluid [20]. In EBC, inflammatory markers, such as cytokines, chemokines and adhesion molecules, can be measured. Increased concentrations of various markers in EBC were found in patients with asthma [10,16-18,21].

### Early lung function measurements

In the past 10 years new lung function techniques became available in young children. Techniques to evaluate airway resistance like the interrupter technique (MicroRint), impulse oscillation, and forced oscillation technique are increasingly applied in young children [22-24]. In contrast to the forced expiration manoeuvres, these measurements are performed during tidal breathing. The measurements are possible in children of 1–2 years and over. However, feasibility increases with age [22]. These techniques are used in children with asthma to assess baseline airway resistance, reversibility on bronchodilators, bronchial hyperresponsiveness, and responses to ICS.

### Response to inhaled corticosteroid treatment

A good responsiveness to ICS is a hallmark of asthma [25]. This response might discriminate between asthmatic and non-asthmatic children. Several international guidelines advocate a trial of ICS in preschool children with recurrent wheeze. However, the diagnostic value of the response to ICS for asthma is not clear in these children.

### Regulatory T-cells

The inflammatory response in asthma is highly complex in which many inflammatory cells are involved. T-helper (Th) cells play a central role in the inflammatory response in asthma, and can be roughly divided in the pro-inflammatory Th2 cells and anti-inflammatory Th1 cells [26-29]. Regulatory T cells (T_reg _cells) inhibit both Th1 and Th2 cells which results in a balance of the immune system. An imbalance between Th2 and Th1 cells occurs in asthma with an increase of Th2, and a decrease of Th1 cells [26,27]. This imbalance might be due to a decrease in amount or function of T_reg _cells [28,29].

### Genetic background

**Figure 1:**
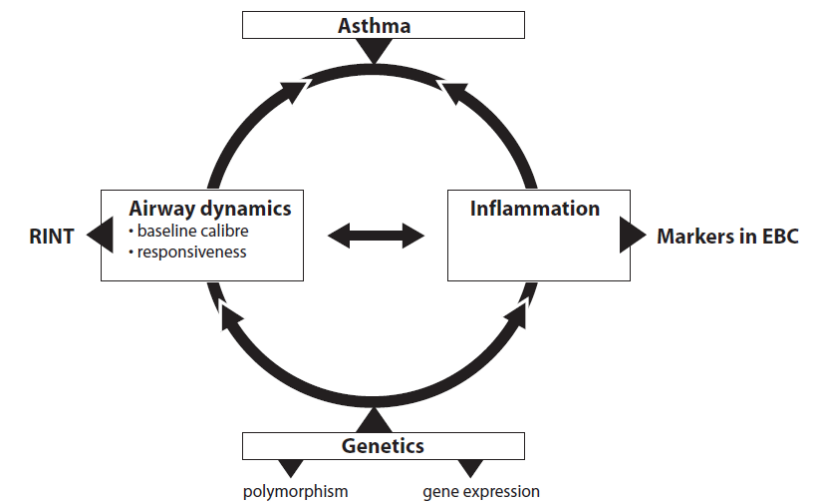
**Relation between genetic background and pathophysiology in asthma**. Central features in the pathophysiology of asthma are chronic airway inflammation and airway (hyper)responsiveness. These features can be measured by markers in exhaled breath condensate and MicroRint, respectively, and are influenced by genetic factors.

Asthma has a multifactorial aetiology in which genetic factors, environmental influences, and their interaction play an important role. Over the last two decades the genetic background of asthma has become increasingly clear through twin studies, and studies in subjects with a family history of atopy and asthma. Around 30 to 100 genes are involved in asthma [30,31]. Although a lot of progress has been made in the field of asthma genetics, the influence of many candidate genes in relation to asthma susceptibility in young children needs to be further defined. Gene polymorphism in the coding sequences of genes may affect the function of a protein. Polymorphisms in the regulatory and promoter sequences of genes may influence the expression characteristics of a gene, making gene expression profiling an important area in asthma research. Linking specific genetic polymorphisms and gene expression profiles to wheezing phenotypes in children and to inflammatory levels in EBC and lung function indices, leads to a better understanding of early pathogenesis of asthma in young children (figure 1). Subsequently, this will identify children with enhanced risk.

### Environmental factors in relation with asthma

The hygiene hypothesis suggests a relation between exposure to microbes in early childhood and the development of allergies and asthma. According to this hypothesis, infections protect against asthma [32,33]. However, this is not in line with increasing evidence that certain infections might also promote asthma development [33]. A unifying concept is still lacking, and the precise relationship between infections and the development of asthma is not clear.

### Hypothesis

The primary hypothesis of the ADEM study (Asthma DEtection and Monitoring study), is that an early asthma diagnosis is possible using non-invasive measurements of biomarkers of airway inflammation and oxidative stress in exhaled breath (condensate), and early lung function measurements (airway resistance). Besides, this study tests the hypothesis that certain gene polymorphisms and gene expression profiles, early infections, and T_reg _cells in blood are related to the development of asthma.

### Aim and research questions

The primary aim of the study is to develop a non-invasive instrument for an early asthma diagnosis in young children. Besides, aetiological factors (such as T_reg _cells, gene polymorphisms, gene expression profiles and infections at early age) are studied in relation to the early development of asthma. This second part of the study has an explorative character.

#### The primary research question is

Which non-invasive inflammatory biomarkers in exhaled breath (condensate) or early lung function indices (baseline airway resistance, response after a bronchodilator) can reliably predict asthma at an early age?

#### The secondary research questions are

1) What are the differences in inflammatory biomarkers and lung function indices between asthmatic and non-asthmatic children?

2) Which of the selected gene polymorphism and/or gene expression profiles are related to asthma susceptibility in young children?

3) Is early colonisation of the airways and intestines related to the development of asthma?

4) What are the differences in amount of T_reg _cells between asthmatic and non-asthmatic children?

5) What is the relation between gene coding and gene expression of inflammatory markers (e.g. genes coding for IL-4, sICAM, IL-13, TNF-α, and ADAM-33), levels of inflammatory markers in EBC, and lung function indices during the development of asthma in young children?

To answer these research questions, markers in different media are measured (table 1).

**Table 1:** Overview of measurements per visit

**Media/method**		**ED I**	**ED II**	**ED III**	**Follow- up**	**Definite diagnosis**
Exhaled breath	Nitric Oxide	●	●	●	●	●
	Volatile organic compounds	●	●	●	●	●
Exhaled breath condensate	Cytokines (IL1a,-2,-4,-5,-6,-10,- 12p70,-13,-18, IFNg, TNFa)	●	●	●	●	●
	Chemokines (MIP1a, MIF, eotaxin, RANTES, IL8, MCP1)	●	●	●	●	●
	Adhesion molecules (sICAM)	●	●	●	●	●
	Nitrate/nitrite	●	●	●	●	●
Blood	White blood cell count, differentiation, number of eosinophils	●				
	Total Immunoglobulin E (IgE), and specific IgE	●				●
	Regulatory T cells	●				
	Gene polymorphism (e.g. in IL-4, IL-13, TNF-alpha, ADAM33)	●				
	Gene expression (e.g. in IL-4, IL-13, TNF-alpha)	●				
	Anti bodies against Mycoplasma en Chlamydia pneumoniae	●				
Saliva	Colonisation of Pneumococcen, Haemophilus (para)influenza, Staphylococcus aureus	●				●
Faeces	Colonisation of E.Coli en Clostridium difficile	●				
Lung function test	Airway resistance (MicroRint) before and after bronchodilator	●	●	●	●	
	Dynamic spirometry (MEFV, FEV_1_, FVC, MEF_50_) before and after bronchodilator					●
	Histamine provocation test					●
Questionnaire	Parental administrated respiratory symptoms (ISAAC)	●	●	●	●	●
	Demographic factors (e.g. smoking, pets)	●	●	●	●	●
	Parental administrated Quality of life (FSII)	●	●	●	●	●

ED I/II/III = Consecutive measurements in early diagnosis phase

## Methods/Design

### Study design

**Figure 2:**
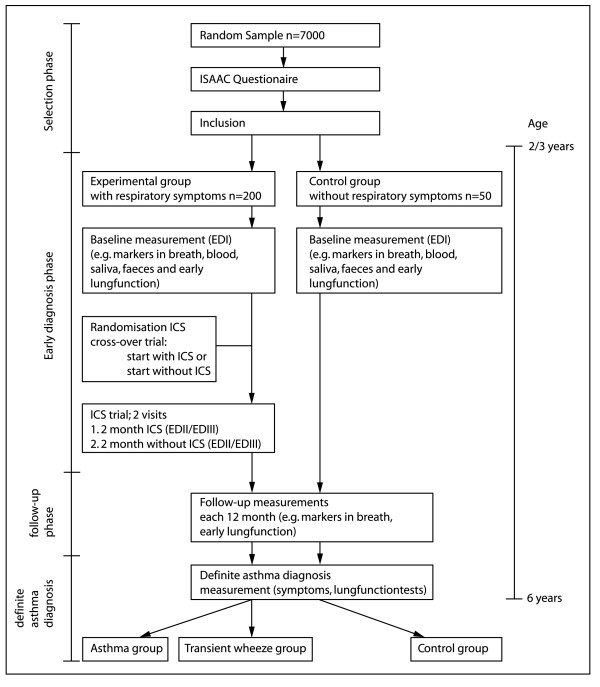
**Study design**. ED I/II/III = Consecutive measurements in Early Diagnosis phase; ICS = Inhalation corticosteroids.

The study design is a long-term prospective case-control study during 4 years. The study consists of four phases: 1) the selection phase; 2) the early diagnosis phase; 3) the follow-up phase; and 4) the definite diagnosis at 6 years (figure 2).

In the *selection phase*, a random sample of children aged 2–3 years of primary care practice and of two cities in Limburg, the Netherlands, receives a standardised questionnaire on respiratory symptoms (ISAAC) [34]. From the results of this questionnaire, a group of 200 children with recurrent asthma-like symptoms (experimental group), and 50 children with no respiratory symptoms (control group) are selected. In the *early diagnosis phase*, a two-month trial with ICS is performed. In addition, repeated measurements of early predictors, like exhaled biomarkers of inflammation/oxidative stress, and lung function tests are assessed. During the *follow-up phase*, the development of respiratory symptoms, lung function indices, and inflammatory biomarkers are studied. At six years of age, a *definite diagnosis *of asthma is made, based on various lung function measurements and current respiratory symptoms ('golden standard'). At this stage, early measurements of (non-invasive) inflammatory biomarkers and lung function measurements are related to the final diagnosis of asthma in order to select the combination of biomarkers which can assess asthma reliably. The study design is described in more detail below.

### Selection phase

Subjects are recruited from two sources. The first source consists of general practices from the Registration Network of Family Practices of the University of Maastricht. This department is used for other studies and has extensive research logistics, including 55 general practitioners and 110,000 patients [35]. In addition, a community-based random sample of children aged 2–3 is selected of two cities in Limburg (Maastricht and Heerlen).

Parents receive information about the study, along with the informed consent form. When parents are willing to participate with their child in the study, they are asked to fill in an internationally standardised questionnaire on respiratory symptoms (ISAAC) [34]. From the results of the ISAAC questionnaire, 200 children with recurrent respiratory symptoms (experimental group) and 50 with no respiratory symptoms (control group) are selected.

#### Experimental group

In total, 200 children aged 2–3 years old with recurrent respiratory symptoms participate in the experimental group. The inclusion criterion for this group is that children experienced at least 2–3 episodes of wheeze during their life, based on the parents-completed ISAAC questionnaire. Exclusion criteria are mental retardation, cardiac anomalies, congenital malformations, other diseases of the lungs/airways, Crohn's disease or rheumatic arthritis, and the inability to perform lung function measurements or exhaled breath collection. The use of ICS is not an exclusion criterion. However, ICS are stopped at least four weeks before the start of each measurement.

#### Control group

In addition, 50 children aged 2–3 years without wheeze and other recurrent respiratory symptoms are selected, based on the parents-completed ISAAC questionnaire. Exclusion criteria are similar as for the experimental group.

After written informed consent, children and parents are invited for a visit to the lung function laboratory. The lung function assistant and/or research physician further evaluates suitability for participation. A questionnaire on demographic data, medical history of the child, family history, day-care attendance, housing, prescribed drug therapy, exposure to pets, and passive smoking is completed.

### Early diagnosis phase

In the early diagnosis phase, repeated measurements of early predictors, including lung function tests and markers of inflammation/oxidative stress in exhaled breath (condensate), blood and faeces are performed in both experimental and control group (table 1). Besides, a controlled cross-over trial with ICS is part of the diagnosis phase for the children in the experimental group. This trial consists of a treatment period of two months with ICS therapy (Beclametasone extra fine two times 100 microgram a day via the Aerochamber^®^), and a two-month period without ICS. Based on randomisation, half the children start with ICS, the other half starts the period without ICS. All other anti-inflammatory medication is stopped. Clinical visits and measurements for the ICS trial occur at 0, 2, and 4 months.

### Follow-up phase

The purpose of the third phase is to monitor development of respiratory symptoms, inflammatory biomarkers, and lung function in both the experimental and control group. At 12-month intervals, each child visits the lung function laboratory for measurements of inflammatory biomarkers and lung function tests. All relevant therapy (e.g. dose and period) is registered. If possible, ICS are stopped four weeks before the measurements. In practices of the participating general practitioners, a computer program for the study is installed. With this program, general practitioners register standardised diagnoses of asthma, atopic dermatitis and allergic rhinoconjunctivitis on-line. Diagnoses are based on international Classification of Health Problems in Primary Care (ICHPPC) definitions.

Children are treated according to the guidelines for treatment of asthma of the Dutch Society of General Practice [36]. These national guidelines approach the international GINA guidelines of asthma diagnosis and treatment [25].

### Definite asthma diagnosis phase

At six years, a definite diagnosis of asthma is made (primary outcome measure and 'golden standard') upon the presence of current asthma symptoms in combination with characteristic lung function abnormalities (reversibility on a beta-2 agonist and/or bronchial hyperresponsiveness).

Airway reversibility is defined as an increase in FEV_1 _of ≥ 9% after 400 microgram of extra fine salbutamol. Bronchial hyperresponsiveness is present when a 20% fall in FEV_1 _is obtained with a provocative concentration of histamine (PC_20_) of 8 mg/ml or less. Early measurements of (non-invasive) inflammatory biomarkers and lung function measurements are related to the final diagnosis of asthma. The definite diagnosis of asthma divides the children in the experimental group into an 'asthma group' and a 'transient wheeze group'.

### Study parameters

As described before, repeated measurements are performed from inclusion until the asthma diagnosis. One hour before each experiment, eating and exercise are not allowed. The parameters that are measured are listed below (table 1).

#### Fractional exhaled Nitric Oxide in exhaled breath

FeNO in exhaled breath is offline collected in a 500 ml inert balloon during tidal breathing. Exhaled breath is collected via a face mask that is connected to a two-way non-rebreathing valve [37]. The valve allows inspiration of NO-free air from a NO-inert reservoir to avoid contamination by ambient NO. To avoid nasal contamination a septum between nose and mouth is placed in the mask. After a washout period of five tidal breaths, an NO-inert bag is connected on the expiratory port of the valve to collect exhaled breath. FeNO levels in the bag are determined by offline sampling using the NIOX^® ^(Aerocrine, Solna, Sweden).

#### Chromatogram of exhaled breath

During tidal breathing, expired air is collected in a 1-litre inert bag by means of the 2-way valve system described above. After collection, the bag is immediately emptied across a small tube with active carbon, with rapid adsorption and stabilisation of volatile markers. A profile of inflammatory biomarkers in exhaled breath is assessed by means of gas chromatography time-of-flight mass spectrometer (GC-TOF-MS) [38].

#### Inflammatory markers in exhaled breath condensate

**Figure 3:**
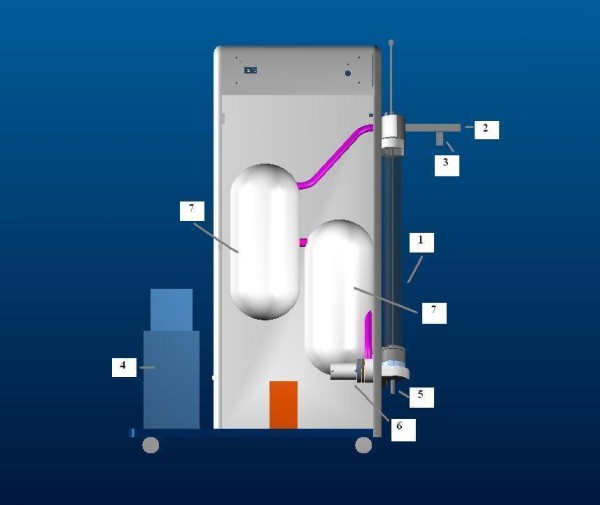
**Schematic representation of the glass closed condenser**. 1. Inclined glass condenser with a moveable plunger. 2. Swan-neck tubing (saliva trap) and two-way non-rebreathing valve, connected to a face mask with separated nose and mouth cavity. 3. Entrance of inspired room air. 4. Cooling unit. 5. Sample vial to collect EBC. 6. Ventilator system for recirculation of non-condensed exhaled breath. 7. Heated (at 37°C) inert Tedlar™ gas sample bag to collect the residual non-condensed exhaled breath.

To collect EBC in young children a special system is designed in close collaboration with the Department of Instrument Development Engineering & Evaluation of the MUMC [19]. Figure 3 shows a schematic representation of this closed glass condenser. In short, children breathe tidally for ten minutes through a mask connected to the two-way non-rebreathing valve. EBC is collected using a cooled double-jacketed glass condenser that is connected to the valve via a tube. The two-way valve and tubing serve as a trap to minimize salivary contamination. Circulating ice water cools the condenser to 0°C. During this procedure small droplets of breath condensate are formed which is collected in a tube. During the procedure, children can watch cartoons. Exhaled breath that does not directly condensate in the condenser is collected in an inert bag that is connected to the condenser. To avoid that the exhaled breath condensates in the bag, the bag is placed in a heated box (37°C). When the child finished the procedure, the exhaled breath that is temporarily collected in the inert bag is conducted through the condenser (recirculation) to increase the amount of condensate. After collection, EBC is rapidly frozen at -80°C using dry ice and is stored at -80°C until analysis. Inflammatory markers (e.g. IFN-gamma, TNF-alpha, Interleukins, sICAM) are assayed by the Luminex^® ^technology (Luminex Corporation, Austin, TX, USA) [39].

#### Polymorphism in inflammatory genes

Saliva is used for DNA collection. DNA is isolated according to the protocol of Oragene (Oragene, Ottowa, Canada). For genotyping matrix-assisted laser desorption/ionization-time of flight (MALDI-TOF) mass spectrometry is used (Sequenom Inc., San Diego, USA). Sequences are evaluated in the ProxSNP and PreXTEND software . The reactions are designed using Sequenom Assay Designer 3.1 software. Genotyping is executed according to the iPLEX method. In short, multiplexed polymerase chain reaction (PCR) is performed in 5 ul volume using the Sequenom PCR kit. Deactivation of unincorporated deoxyribonucleotide triphosphates (dNTPs) is achieved using shrimp alkaline phosphatase. The iPLEX reagent kit carries out primer extension. To remove residual salt from the reactions a cation exchange resin is added. Approximately 15 nl of the primer extension reaction are loaded onto a matrix pad of a SpectroCHIP (Sequenom). MassARRAY Compact Analyzer is used to analyse the SpectroCHIPs (Sequenom). The PCR primer and extension primer sequences are available on request. The genotyping calls are made using Typer Analyzer 4.0 software. The SNP's that are studied are selected from Pubmed in combination with the HapMap database . Inclusion of genes for SNP's analysis is based on the following criteria: association with asthma based on biomedical literature, a functional difference between the variant allele and the wild-type allele and a minor allele frequency of at least 5% in the (asthmatic) population. Genes selected for analysis are: Fillagrine, IL-4, IL-10, IL-13, TNF-alpha, sICAM, ADAM33, Toll-like receptors and ORMDL3.

#### Venous blood sampling

At the first visit of the study, venous blood is sampled for:

1. White blood cell count, differentiation, number of eosinophils;

2. Total Immunoglobulin E (IgE) and specific IgE for pollen, cats, dogs, house dust mite, Aspergillus Fumigatus (Pharmacia, Uppsala, Sweden);

3. Presence of T_reg _cells (described below);

4. Gene-expression profiles of relevant markers (described below);

5. Infection serology (Mycoplasma, Chlamydia).

In addition, total and specific IgE are also determined at the last visit.

#### Regulatory T-cells

T_reg _cells were quantified in the circulation by flow cytometry. The phenotype of the T_reg _was defined as being positive for CD3, CD4, and CD25 (IL-2Rα), while being negative for CD127 (IL-7Rα). Cells with this phenotype have been shown to be positive for FoxP3 [40], a transcription factor which is closely related to the suppressive function of T_reg_.

#### Gene expression of markers of inflammation

In addition to gene polymorphisms, gene expression markers of inflammation are determined in the early diagnosis phase of the study. Total RNA from venous blood is isolated that is used to generate cDNAs by poly-A and random priming reverse transcription. The cDNAs are stored and used for the production of copy RNAs pools that can be used to hybridize to custom made arrays of genes. Hybridization of the arrays enables us to select genes with an up- or down-regulated expression in children with asthma. The array hybridization is used only as a primary indicator and the results are validated by real-time quantitative PCR. Inclusion of genes for gene expression is based on the same criteria as the inclusion of genes for SNP's analysis and on an altered gene expression in asthma suggested in the literature. Genes studied are the same genes as for polymorphisms (described above). In addition eotaxin, RANTES, MIF, MIP1alpha, GST, heme oxygenase, catalase, superoxide dismutase, NOS1, STAT6, NF-kappa B, chitinase and keratins are studied.

#### Infection serology

At the start and at the end of the study, a nose- and throat swab is collected to analyse possible colonisations of Pneumococcen, Haemophilus (para) influenzae, and Staphylococcus aureus. At the first visit, antibodies of Chlamydia and Mycoplasma pneumoniae are analysed in venous blood, using ELISA. In addition, faeces are tested for E. coli and Clostridium difficile.

#### Lung function tests of airway resistance

Measurements of airway resistance are performed by means of the MicroRint (Micro Medical, Rochester Ltd, UK) [22]. While children are sitting in an upright position, they are asked to breathe tidally through a facemask. Seven airflow interruptions are made on the peak flow of expiration. The median MicroRint value together with the flow and pressure curves are displayed. The median MicroRint value of at least five successful interruptions is used for analysis. Thereafter, 300 microgram of extra fine salbutamol is inhaled via the Aerochamber^®^. After 15 minutes, MicroRint measurements are repeated to assess the reversibility to a beta-2 agonist.

#### Dynamic spirometry, bronchial hyperresponsiveness and reversibility

At the end of the study, additional lung function tests are performed like described before [35]. Maximal expiratory flow volume curves (MEFV) curves are assessed in each child by means of the Flowscreen^® ^(Jaeger, Wuerzburg, Germany). The highest forced expiratory volume in one second (FEV_1_), forced vital capacity (FVC), and maximal expiratory flow at 50% FVC (MEF_50_) of three technically satisfactory MEFV curves is used for analysis. After three baseline MEFV curves, an aerosol of buffered saline is inhaled, followed by aerosols of histamine acid phosphate in doubling concentrations from 0.03 to 16 mg/ml at five minute intervals. Dynamic spirometry is repeated once after 30, and after 90 seconds following each inhalation. The inhalations of histamine are discontinued in case of a 20% fall in FEV_1 _(PC_20_) or when 8 mg/ml histamine has been administered. Thereafter, 400 microgram of extra fine salbutamol is inhaled. After 15 minutes, three MEFV curves are assessed in a comparable way. The change in FEV_1 _is expressed as a percentage of the predicted value.

#### Questionnaires on respiratory symptoms and quality of life

Presence of cough, breathlessness, and wheezing are registered according to the internationally standardised ISAAC questionnaire [34]. Besides, a questionnaire designed for preschool children to record patterns of wheeze and other respiratory symptoms is completed by parents [41]. In addition, medical history in relation to respiratory symptoms is gained by a physician [42]. The quality of life in children is measured by parent administered general health related quality of life (FSII) questionnaires.

### Samples size calculations

In a population of infants with recurrent asthma-like symptoms, the prevalence of asthma at 6 years is known to be 30% [1]. In this study, this results in at least 50 asthmatic children given the 200 children with recurrent respiratory symptoms aged 2–3 years at the start, and a 10% drop-out rate. The standard error of the sensitivity given a chance on a positive test result of 0.8 will be 4.0%. The standard error of the specificity with 150 children without disease will be 3.0% given a chance on a positive test result of 0.8. If the positive predictive value of the test is 0.7 and the negative predictive value 0.2 in a population of N = 200, the statistical power for the relation between the test and the definite diagnosis of asthma is 98%.

### Data collection

The collected data are checked and cleaned by the centre for data and information management of Maastricht University (MEMIC). All data are stored in a database at MEMIC.

### Data analysis

The data are analysed using quantitative statistics. Normally distributed data are expressed as mean and standard error. Not normally distributed data are expressed as median with interquartile ranges. Different analysis is performed concerning the primary research question and secondary research questions.

#### Statistics of the primary research question

The primary research question is whether non-invasive inflammatory biomarkers in exhaled breath and lung function indices can reliably predict asthma at an early age. For this purpose, biomarkers collected in the experimental group during the early diagnosis phase are used. These parameters are related to the definite diagnosis of asthma by means of multiple logistic regression analysis, and discriminant analysis. Receiver Operating Characteristic (ROC) curves and area under the curves (AUC) are calculated.

#### Statistics of the secondary research questions

To answer the second research questions, data from both experimental group and control group are used. Differences in parameters between the three groups (asthma, 'transient wheezers', and controls) are tested with analysis of variance (one-way ANOVA or Kruskal-Wallis test in case of parametric and non-parametric data, respectively). For normally distributed data, Student's t-tests are used for further analysis. Mann-Whitney U tests are used to test for differences among non-parametric data. Differences are defined as significant when p < 0.05.

### Ethics

Ethical approval is obtained from the Dutch National Medical Ethical Committee (CCMO). All parents gave written informed consent. At the end of the study all parents are informed about the personal primary outcome measure (asthma diagnosis) of their child, and general results of the study. The study protocol is extensively studied by the funding organizations: the Dutch Asthma Foundation, Stichting Astma Bestrijding, and Maastricht University Medical Centre. This study is registered by clinicaltrial.gov (NCT 00422747).

## Discussion

We presented the protocol of the ADEM study that aims at an early asthma diagnosis in young children using non-invasive biomarkers of airway inflammation and early lung function measurements. The design of the study is a prospective, case-control study in 200 children with recurrent respiratory symptoms and 50 control subjects with no respiratory symptoms. Our primary hypothesis is that an early asthma diagnosis is possible using non-invasive measurements of inflammatory biomarkers in exhaled breath (condensate), and lung function tests. Besides, aetiological factors including gene polymorphisms, gene expression profiles, T_reg _cells and microbial colonisation of airways and intestines are studied in relation to the development of asthma.

The development of a new diagnostic tool for asthma at an early age, based on non-invasive inflammatory biomarkers in exhaled breath, and lung function can lead to an early asthma diagnosis. This will result in better treatment and probable a better prognosis of asthma in children. Moreover, exclusion of asthma at an early age prevents over-treatment in the children with transient wheezing, which will reduce possible side effects and economic costs. The design and setting of the ADEM study is ideally suited to study the relation between different parameters that are measured in early life, and the susceptibility for asthma. This will increase insight in, for example, the relation between the genetic background and the pathophysiology of asthma in young children.

There are several critical success factors to be mentioned.

The study performs measurements in children aged 2–6 years old. Although the measurements are not invasive, the question arises whether the measurements are feasible in these young children. In a previous study in 70 preschool children, we established a success rate of EBC measurements of 83% using the same condenser [19]. With respect to early lung functions measurements, Merkus et al. found a feasibility of 88% of MicroRint measurements in children aged 2 years and over [22]. We hope to achieve success rates of the measurements of at least 90% taken into account the non-invasive character of the measurements, and our experience and learning curve in the field of measurements of exhaled breath (condensate) and lung function tests in children [19,35].

A trial with ICS is part of the diagnosis phase for the children in the experimental group. A trial period with ICS is often advocated but the precise diagnostic value is not clear from earlier studies [25]. The children in the experimental group consist of children who experienced at least 2–3 episodes of wheeze during their life. This can imply that some children are symptom free at the start of the ICS trial. Therefore, we expect a lower compliance in the group children without current respiratory symptoms. Compliance will be measured by weighting the ICS inhalators before and after the trial.

Children in this study are followed up for 3–4 years. This long-term study can induce lost to follow-up, which is dependent on compliance and the involvement of the parents with the study. In our power analysis we calculated a lost to follow-up of 10%. Other studies of our research group with a comparable design had a similar drop-out rate [43].

The field of asthma genetics is evolving rapidly and genes involved in asthma are discovered on a regular basis. Therefore, selection of genes in this study is an ongoing process and genes may be added if found relevant in the literature.

In this study various parameters are measured. Because of the large number of parameters that are measured, it is important to distinguish the primary outcome measure from the aetiological factors that are studied. The results of the aetiological part of this study have a more exploring character and should be tested more extensively in future research.

## Conclusion

As an early diagnosis of asthma is currently difficult, both under-diagnosis and under-treatment of asthmatic children as well as over-treatment of transient wheezers occur frequently. So far, assessments of inflammatory biomarkers play a minor role in the diagnosis of asthma. The development of a new diagnostic tool for asthma at an early age, based on non-invasive inflammatory biomarkers in exhaled breath and early lung function measurements is a promising technique. These new diagnostic techniques are applied in this study and probably make an early diagnosis of asthma possible. This will result in earlier and better treatment of childhood asthma.

## Abbreviations

ADAM33: A Disintegrin And Metallopeptidase domain 33; dNTPs: Deoxyribonucleotide triphosphates; EBC: Exhaled Breath Condensate; FeNO: Fractional exhaled Nitric Oxide; FEV_1_: Forced expiratory volume in one second; FVC: Forced vital capacity; GC-TOF-MS: Gas chromatography time-of-flight mass spectrometer; GST: gluthathiontransferase; ICS: Inhalation corticosteroids; IFN-gamma: Interferon-gamma; IgE: Immunoglobulin E; IL-4/IL-10/IL-13: Interleukin 4/Interleukin 10/Interleukin 13; MEFV: Maximal expiratory flow volume curves; MEF_50_: Maximal expiratory flow at 50%; MIF: Macrophage migration Inhibitory Factor; MIP1-alpha: Macrophage Inflammatory Protein 1 alpha; NF-kappaB: Nuclear Factor kappa-light-chain-enhancer of activated B cells; NOS1: Nitric Oxide Synthase 1; ORMDL3: Orosomucoid 1-like 3, ORM1-like 3; PC_20_: Provocative concentration of histamine leading to a 20% fall in FEV; PCR: Polymerase chain reaction; RANTES: Regulated upon Activation Normal T-cell Expressed, and Secreted; SBE-CGE: Single Base Extension combined with Capillary Gel Electrophoresis; sICAM: soluble Intercellular Adhesion Molecule; SNP's: Single Nucleotide Polymorphisms.

## Competing interests

The authors declare that they have no competing interests.

## Authors' contributions

KDGK is the investigator of the study and wrote the manuscript. EMMK is the second investigator of the study and contributed to the writing of the manuscript. AJN is a Master student in Health Sciences and contributed to the acquisition of data. QJ, CPS and ED are supervisors and applicants of the grants for the study. They developed the study and contributed to the study coordination and the writing of the manuscript. All authors read and approved the final manuscript.

## Pre-publication history

The pre-publication history for this paper can be accessed here:


